# Hospitalisation rate and mortality among people with and without diabetes during the COVID-19 pandemic year 2020

**DOI:** 10.1007/s10654-022-00865-6

**Published:** 2022-06-08

**Authors:** Maria Narres, Heiner Claessen, Tatjana Kvitkina, Joachim Rosenbauer, Maria Scheider, Stephan Morbach, Andrea Icks

**Affiliations:** 1 Institute for Health Services Research and Health Economics, German Diabetes Centre (DDZ), Leibniz Centre for Diabetes Research at Heinrich-Heine-University Düsseldorf, Auf´m Hennekamp 65, 40225 Düsseldorf, Germany; 2 Institute for Health Services Research and Health Economics, Center for Health and Society, Faculty of Medicine, Heinrich Heine University Düsseldorf, Düsseldorf, Germany; 3 German Center for Diabetes Research (DZD), Munich-Neuherberg, Germany; 4 Institute for Biometrics and Epidemiology, German Diabetes Center (DDZ) Leibniz Center for Diabetes Research at Heinrich Heine University Dusseldorf, Düsseldorf, Germany; 5 Department of Health Management, AOK Rheinland/Hamburg – die Gesundheitskasse, Düsseldorf, Germany; 6 Department of Diabetes and Angiology, Marienkrankenhaus, Soest, Germany

## Abstract

Most studies reported reduced health care use among people with diabetes during the COVID-19 pandemic. This may be due to restricted medical services or people avoiding health care services because they fear being infected with COVID-19 in health care facilities. The aim of our study was to analyse hospitalisation and mortality in people with and without diabetes in Germany during the COVID-19 pandemic year 2020 compared to 2017–2019. The data were sourced from a German statutory health insurance company covering 3.2 million people. We estimated age-sex standardised rates of mortality, all-cause hospitalisation, hospitalisation due to coronary heart disease (CHD), acute myocardial infarction (AMI), stroke, diabetic foot syndrome (DFS), and major and minor amputations in people with and without diabetes. We predicted rates for 2020 using Poisson regression based on results from 2017–2019 and compared these with the observed rates.In people with diabetes, the hospitalisation rate for major amputation was significantly increased, while all-cause hospitalisation rate and hospitalisation due to CHD, AMI and DFS were significantly decreased compared to the previous period. Moreover, we found a significantly increased mortality and hospitalisation rate for minor amputation in people without diabetes while all-cause hospitalisation and hospitalisation due to CHD and AMI was significantly lower during the COVID-19 pandemic year 2020.We observed changes in health care utilisation and outcomes during the COVID-19 pandemic compared to previous years in people with and without diabetes. Concerning diabetes care, the increase of hospitalisations due to amputation in people with diabetes with a simultaneous reduction in DFS needs special attention.

People with diabetes have a high risk of hospitalisation and mortality due to COVID-19 (coronavirus) infection [1]. However, a number of studies reported reduced health care use among people with non-communicable diseases, including diabetes, during the COVID-19 pandemic [2]. This may be due to restricted medical services or avoidance of health care use for fear of infection in health care facilities [3]. Nevertheless, the extent of this change and its impact on health outcomes in people with and without diabetes is unclear. The aim of our study was to analyse hospitalisation and mortality in people with and without diabetes in Germany during the COVID-19 pandemic year 2020 compared to 2017–2019.

## Methods

We used data from one German statutory health insurance company (Allgemeine Ortskrankenkasse (AOK) Rheinland/Hamburg) covering 3.2 million insured people. We included data from all individuals who had continuously been insured for at least two subsequent calendar years between 2016 and 2020. The diabetes status of all people including incident cases was defined for each calendar year according to an established algorithm [https://www.wido.de/fileadmin/Dateien/Dokumente/Publikationen_Produkte/Gesundheitsatlas/wido_int_gesundheitsatlas_deutschland_1119.pdf]. We estimated age-sex standardised mortality rates, rates for all-cause hospitalisation, and hospitalisation rates due to coronary heart disease (CHD), acute myocardial infarction (AMI), stroke, diabetic foot syndrome (DFS), and major and minor amputations in people with and without diabetes using the German population 2019 as standard population. For each individual, hospitalisation per calendar year was defined as at least one hospitalisation in the respective year. We predicted rates for 2020 using Poisson regression based on age-sex standardised rates from 2017 to 2019 and compared these with the observed rates with estimation of relative risks (RR).

## Results

The average prevalence of diabetes during the study period was 10.3%. In 2020, 42,117 people died (diabetes: 14,459) and 615,015 people had at least one hospitalisation (diabetes: 134,040). The observed hospitalisation rates in 2020 were significantly lower than predicted in people with diabetes for all-cause hospitalisation (RR 0.84 [95% confidence interval 0.84–0.85], p < 0.001), hospitalisation due to CHD (RR 0.88 [0.85–0.92], p < 0.001), AMI (RR 0.89 [0.83–0.95], p = 0.007) and DFS (RR 0.77 [0.71–0.83], p < 0.001), Table 1). In contrast, the hospitalisation rate due to major amputation was significantly higher than predicted (RR 1.36 [1.12–1.65], p = 0.002), Table 1). We did not find significant changes regarding mortality, stroke and minor amputation.

**Table 1:** Mortality and hospitalisation rate in people with and without diabetes in 2020 compared to 2017–2019

		Diabetes			No Diabetes			
		Observed rate ^a^ (O)	Predicted rate ^a^ (E)	RR O/E	Observed rate ^a^ (O)	Predicted rate ^a^ (E)	RR O/E	
Mortality		2110.7 (2059.7-2161.7)	2150.1 (1986.7–2327.0)	0.98 (0.95–1.02)	1480.1 (1460.8-1499.3)	1424.4 (1378.8-1471.5)	1.04 (1.02–1.05)^*^	
Any hospitalisation		37555.1 (37020.1-38090.0)	44598.5 (43857.9-45351.5)	0.84 (0.84–0.85)^*^	20358.3 (20297.2-20419.4)	23544.9 (23361.6-23729.7)	0.86 (0.86–0.87)^*^	
Hospitalisation due to								
- Coronary heart disease		1468.2 (1427.7-1508.8)	1668.3 (1490.7-1866.9)	0.88 (0.85–0.92)^*^	607.0 (595.4-618.5)	687.4 (647.9-729.3)	0.88 (0.86–0.90)^*^	
- AMI ^b^		497.3 (474.2-520.3)	561.1 (543.6–579.2)	0.89 (0.83–0.95)^*^	221.6 (214.6-228.6)	232.2 (228.4–236.0)	0.95 (0.92–0.99)^*^	
- Stroke		532.7 (510.4–555.0)	551.4 (522.2-582.3)	0.97 (0.90–1.03)	300.6 (292.4-308.8)	298.9 (291.6-306.3)	1.01 (0.97–1.04)	
- DFS ^c^		380.5 (350.9–410.0)	494.7 (391.6-624.9)	0.77 (0.71–0.83)^*^	----	----	----	
- Major amputation		79.2 (69.3–89.1)	58.2 (48.5–69.9)	1.36 (1.12–1.65)^*^	17.8 (15.8–19.8)	15.9 (13.9–18.1)	1.12 (0.98–1.28)	
- Minor amputation		209.9 (193.6-226.1)	219.8 (180.3-267.9)	0.95 (0.86–1.07)	22.6 (20.3–25.0)	19.7 (15.7–24.6)	1.15 (1.02–1.30)^*^	

* p-value < 0.05

^a^ per 100,000 person years, age-sex standardised using the German population 2019 as standard population, 95% confidence interval in brackets

^b^ Acute myocardial infarction

^c^ Diabetic foot syndrome

Rates observed in people without diabetes were significantly lower than predicted for all-cause hospitalisation (RR 0.86 [0.86–0.87], p < 0.001), hospitalisation due to CHD (RR 0.88 [0.86–0.90], p < 0.001) and AMI (RR 0.95 [0.92–0.99], p = 0.012), while mortality (RR 1.04 [1.02–1.05], p < 0.001) and hospitalisation rates due to minor amputations (RR 1.15 [1.02–1.30], p = 0.020) were significantly higher than predicted. Hospitalisation rates for stroke and for major amputation did not significantly differ from predicted rates. Age and sex stratified analysis in both people with and without diabetes revealed that findings regarding mortality, AMI and minor amputation were predominantly explained by changes in the older age groups. Increased rates of major amputation in people with diabetes were predominantly identified amongst the male population. The results for all other outcomes were consistent in age and sex strata (data not shown).

## Discussion

Our study found a significantly lower hospitalisation rate than predicted for all-cause hospitalisation as well as hospitalisation due to CHD and AMI in both people with and without diabetes during the COVID-19 pandemic. In contrast, we observed an increased hospitalisation rate due to major amputation in 2020 in the population with diabetes. In the population without diabetes, this rate was not increased. The increase of hospitalisations due to amputation in people with diabetes with a simultaneous reduction in DFS is in line with results of other studies and needs special attention [3–5]. A number of underlying reasons can be assumed, including restricted medical services during the COVID-19 pandemic, fear of COVID-19 infection, refusal to attend clinics, unwillingness to be admitted to hospital, and inadequate diabetes self-management due to demotivation when socially distancing coupled with lacking or limited medical support. The increase of observed hospitalisations for minor amputations in people without diabetes might also be explained by reduced medical care during the COVID-19 pandemic.

A limitation of this study is that the data are based on a regional statutory health insurance company. Thus, future nationwide studies in this field are required.

## Data Availability

The data that support the findings of this study are available from the authors upon reasonable request and with permission of the AOK Rheinland/Hamburg.
